# Monocyte to high-density lipoprotein cholesterol ratio predicts poor outcomes in ischaemic heart failure patients combined with diabetes: a retrospective study

**DOI:** 10.1186/s40001-023-01451-6

**Published:** 2023-11-08

**Authors:** Qiuyu Li, Xiaolong Lin, Xiaowen Bo, Fanqi Li, Siyuan Chen, Xuguang Miao, Donghui Zhao, Jinghua Liu, Qian Fan

**Affiliations:** Center for Coronary Artery Disease, Beijing Anzhen Hospital, Capital Medical University, and Beijing Institute of Heart, Lung, and Blood Vessel Diseases, Beijing, 100029 China

**Keywords:** MHR, Monocyte, HDL-C, Ischaemic heart failure, MACE

## Abstract

**Background:**

The prevalence of ischaemic heart failure (HF) continues to increase. Diabetes mellitus (DM) concomitant with ischaemic HF increases the risk of major adverse cardiovascular events (MACEs). As a promising predictor for cardiovascular diseases, the predictive value of the monocyte to high-density lipoprotein cholesterol ratio (MHR) for MACE in the ischaemic HF with DM cohort has never been investigated before.

**Objective:**

We aimed to investigate the MHR as a predictor for MACE in ischaemic HF patients with DM who underwent percutaneous coronary intervention (PCI).

**Methods:**

This observational study enrolled 1049 patients with ischaemic HF and DM undergoing PCI from June 2017 to June 2019. The baseline data were collected. MACEs, including all-cause mortality, nonfatal myocardial infarction, and any revascularization, were recorded within the 36-month follow-up. The characteristics and incidence of MACE were analysed in four groups stratified by the quartiles of MHR. The hazard ratio for MACE was analysed with Cox regression models. The incidence of MACE in the four groups was evaluated by Kaplan‒Meier survival analysis. Restricted cubic spline analysis was performed to determine the nonlinear correlation between the MHR and MACE.

**Results:**

After the 36-month follow-up, 407 patients (38.8%) experienced MACEs. The incidence of MACE was significantly higher among patients in the upper MHR quartile than among those in the lower MHR quartiles (23.4% vs. 36.0% vs. 41.4% and 54.6%; *P* < 0.001, respectively), which was consistent with the Kaplan‒Meier survival analyses (*P* < 0.0001). A multivariate Cox regression model showed that the MHR was an independent risk factor for MACE after variables were adjusted (adjusted HR: 2.11; 95% CI 1.47–3.03; *P* < 0.001). Its predictive effects on MACE showed no interaction with hypercholesterolemia (*P* > 0.05).

**Conclusion:**

The MHR was a significant and independent predictor of MACEs in ischaemic HF patients with DM undergoing PCI.

**Supplementary Information:**

The online version contains supplementary material available at 10.1186/s40001-023-01451-6.

## Introduction

Heart failure (HF) has remained a rising global pandemic with a global prevalence of more than 37.7 million individuals currently [1]. HF is usually associated with increased major adverse cardiovascular events (MACEs), which affects the expected lifespan and brings a huge burden to the health care system [2].

Ischaemic HF accounts for nearly 60–70% of HF cases. Coronary artery disease (CAD) remains the most common cause of ischaemic HF [3]. Patients with ischaemic HF often encounter diabetes mellitus (DM). They share similar metabolic risk factors and independently increase the risk for the other. The prevalence of DM in HF cohorts ranges from 10 to 47%, which is markedly higher than the prevalence in the general population [4–7]. On the other hand, the incidence of HF in individuals with DM was 1.08–6.76 times that in individuals without DM according to many large-scale cohort studies [8–11]. In addition, HF patients with DM have a higher incidence of MACEs than the nondiabetic HF population [8]. In population-based studies, HF concomitant with DM increases the risk of mortality in both hospitalized and ambulatory patients [12, 13]. Therefore, an effective prognostic factor for MACE in ischaemic HF patients with DM is of great value for clinical management.

Whether percutaneous coronary intervention (PCI) improves the clinical outcomes of ischaemic HF remains ambiguous. Some studies found that PCI in patients with complex CAD and left ventricular systolic dysfunction may reverse LV remodelling and improve clinical outcomes [14, 15]. Patients with ischaemic HF subjected to revascularization showed a lower rate of cardiac mortality by 61% in the first 5 years [15]. However, the REVIVED-BCIS2 study demonstrated that compared to optimal medical therapy, PCI in patients with a left ventricular ejection fraction (LVEF) < 35% and myocardial viability did not reduce the rate of mortality [16, 17]. Therefore, the prognosis of ischaemic HF patients undergoing PCI still needs further investigation.

The monocyte to high-density lipoprotein cholesterol (HDL-C) ratio (MHR) is one of the systemic inflammatory indices. The MHR has been proven to have clinical value in the prognosis of CAD after PCI [18–27]. However, in the field of ischaemic HF induced by CAD, the investigation of MHR is limited. The two observational retrospective studies showed that the MHR had potential value in the clinical evaluation of patients with HFpEF and chronic HF [28, 29]. Additionally, previous studies have explored cardiovascular disorders combined with DM cohorts, such as non-ST-segment elevation acute coronary syndrome [24], subclinical left cardiac remodelling and dysfunction [30], and arterial stiffness [31]. All of them showed that the MHR is associated with the occurrence and outcome of certain cardiovascular disorders. However, the application value of the MHR in an ischaemic HF combined with DM cohort has never been assessed before.

Therefore, we carried out a retrospective cohort study to identify the prognostic effect of the MHR on MACE in patients with ischaemic HF with concomitant DM undergoing PCI. The predictive value of the MHR may be more pronounced, offering a potential noninvasive approach for prognostic assessment. This research endeavour is expected to provide novel MHR applications in clinical practice and serve as a basis for developing personalized therapeutic strategies.

## Methods

### Ethics declarations

The Ethics Committee of Beijing Anzhen Hospital, Capital Medical University, and Beijing Institute of Heart, Lung, and Blood Vessel Diseases, Beijing, China approved this study. Informed consent was obtained from all participants in this analysis. All procedures performed in this study involving human participants were in accordance with the Declaration of Helsinki (Registry number: No. 2022235X).

### Study population

**Fig. 1:**
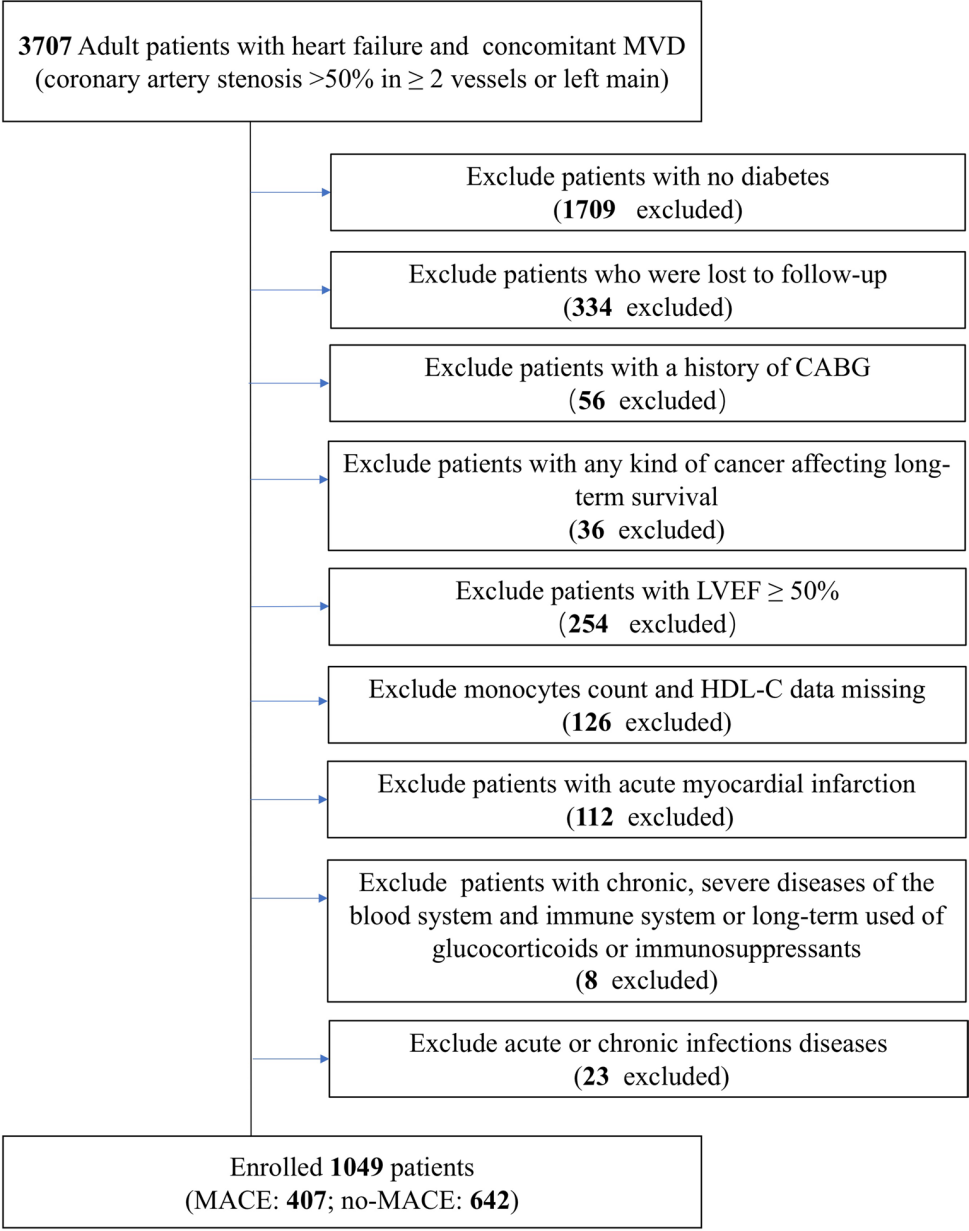
Flow chart of participant inclusion. MVD, multiple vessel disease; CABG, coronary artery bypass graft; LVEF, left ventricular ejection fraction; HDL-C, high-density lipoprotein cholesterol; MACE, major adverse cardiovascular events

This was a single-centre, observational, retrospective cohort study at Beijing Anzhen Hospital. Our patients from mainland China with the primary diagnosis for this study were ischaemic HF and DM undergoing elective PCI from June 2017 to June 2019 at inclusion. The diagnostic criteria of ischaemic HF were as follows [32]: (1) left ventricular failure, congestive HF, cardiac insufficiency, diastolic HF, or HF, unspecified based on the International Classification of Diseases (ICD) 10th revision (I50.106, I50.001, I50.90, I50.919, I50.905, or I50.911). (2) MVD [left main (LM) disease or coronary artery stenosis > 50% in ≥ 2 vessels]. The diagnosis criteria of DM were according to the 1999 criteria of the World Health Organization: fast blood glucose > 7.0 mmol/L or 2‐hour postprandial glucose > 11.1 mmol/L or already diagnosed with DM by the physicians according to the medical history. A total of 3707 adult patients with HF and concomitant MVD undergoing elective PCI in our hospital were enrolled in this cohort. The exclusion criteria of this study were as follows: (1) patients with no DM; (2) patients lost to follow-up; (3) history of coronary artery bypass grafting; (4) any kind of cancer affecting long-term survival; (5) LVEF ≥ 50%; (6) absolute monocyte count and high-density lipoprotein cholesterol data missing; (7) acute myocardial infarction (MI); (8) chronic, severe diseases of the blood system and immune system or long-term use of glucocorticoids or immunosuppressants; and (9) acute or chronic infectious diseases (such as pneumonia, infective endocarditis, or tuberculosis). Ultimately, 1049 patients were included in the final analysis (Fig. 1).

### Data collection

Demographics, vital signs, body mass index (BMI), comorbidities, medical history, New York Heart Association (NYHA) class, echocardiography, angiographic characteristics, procedural results, laboratory examinations, and medication use were collected from the hospital information recording system. Demographics included age, sex. Vital signs included systolic blood pressure (SBP), diastolic blood pressure, and heart rate. Comorbidities included hypertension, hypercholesterolemia, renal insufficiency, and atrial fibrillation. Medical history included prior MI, prior PCI, and prior stroke. Echocardiography data included left ventricular end systolic dimension (LVDs), left ventricular end diastolic dimension (LVDd), and LVEF.

Angiographic characteristics included LM disease, three-vessel disease, chronic total occlusion, diffuse lesion, in-stent restenosis, synergy between PCI with taxus, and cardiac surgery (SYNTAX). The lesion characteristics were defined as follows: (1) LM disease: an angiographically estimated stenosis > 50% or a fractional flow reserve < 0.80 in the LM coronary artery ostium, mid-shaft, or distal bifurcation. (2) Three‑vessel disease: more than two main coronary branches (vessel diameter ≥ 2 mm) with an extent of stenosis ≥ 50%. (3) Chronic total occlusion lesion: lesion with complete obstruction [thrombolysis in myocardial infarction (TIMI) flow grade 0 lasting longer than 3 months, which was judged from the medical history or coronary angiogram results. (4) Diffuse lesion: a single stenotic lesion with a length of ≥ 20 mm. (5) In-stent restenosis: stenosis of ≥ 50% occurring in the segment inside the stent, 5 mm proximal or distal to the stent [33]. The severity of coronary artery lesions was quantified by the synergy between the PCI and SYNTAX score. The SYNTAX score was calculated for each participant using the SYNTAX score algorithm (www.syntaxscore.com) [34]. Procedural results included target vessel territories: LM, left anterior descending artery (LAD), left circumflex artery (LCX), and right coronary artery (RCA), complete revascularization, and the number of stents. PCI strategies were determined and performed by at least two experienced interventional cardiologists and were in accordance with current practice guidelines in China [35].

Laboratory examinations included glucose, glycosylated haemoglobin A1c (HbA1c), glycated albumin, B-natriuretic peptide (BNP), fibrinogen (FBG), D-dimer, International Normalized Ratio (INR), total cholesterol (TC), triglyceride, low-density lipoprotein cholesterol (LDL-C), HDL-C, total bilirubin, direct bilirubin, alanine transaminase(ALT), aspartate transaminase (AST), alkaline phosphatase (ALP), gamma-glutamyl transferase (GGT), creatinine, blood nitrogen urea, estimated glomerular filtration rate (eGFR), uric acid, sodium, potassium, calcium, magnesium, white blood cell, haemoglobin, platelet, high-sensitivity C-reactive protein (hs-CRP), and MHR. Medications included aspirin, clopidogrel, ticagrelor, statins, ezetimibe, angiotensin-converting enzyme inhibitor (ACEI), angiotensin receptor blocker (ARB), beta-blockers, calcium channel blocker (CCB), loop diuretics, thiazide diuretics, spironolactone, tolvaptan, sacubitril/valsartan, metformin, alpha‑glucosidase inhibitor, sulfonylurea, sodium-dependent glucose transporters 2 (SGLT2) inhibitor, dipeptidyl peptidase inhibitor 4 (DDP4) depressor, insulin, warfarin, factor Xa inhibitors, and factor IIa inhibitors.

### Grouping and endpoints

The MHR was determined as the absolute monocyte count (× 10^9^/L) divided by HDL-C (mmol/L). All patients were stratified into four groups according to MHR quartiles: Quartile 1 (MHR < 0.33[× 10^9^/mmol]), Quartile 2 (0.33 ≤ MHR < 0.46[× 10^9^/mmol]), Quartile 3 (0.46 ≤ MHR < 0.62[× 10^9^/mmol]), and Quartile 4 (MHR ≥ 0.62 × 10^9^/mmol]). After baseline PCI, the outcome data of patients were collected at 3, 6, 9 and 12, 24, and 36 months after PCI by trained physicians in the outpatient follow-up centre. The collected information was confirmed according to the medical records when necessary. MACE was defined as the combination of all-cause mortality, nonfatal MI, and any revascularization. The definition of MI was determined by the fourth universal definition of MI [36]. Any revascularization was defined as coronary revascularization due to any reason. The primary endpoint was MACE, and the secondary endpoint was any of the components of the defined MACE. In patients who had multiple adverse outcomes during follow-up, we first selected all-cause mortality, followed by nonfatal MI and any revascularization. Only the first incidence was analysed when events occurred more than once. The present study lasted until June 2022.

### Statistical analysis

Stata software (StataCorp LLC, TX, USA, ver. 15.0) and R software (R-project®, Vienna, AUS, ver. 4.2.1) were used for analysis. *P* < 0.05 was considered statistically significant. Normally distributed continuous variables are presented as the mean ± SD. The abnormally distributed continuous variables are presented as the median (25th, 75th). Categorical variables are presented as numbers (percentages). *P* values were calculated using analysis of variance. Numerical variable comparisons among the groups were performed by ANOVA (for normally distributed continuous data) and the Kruskal‒Wallis test (for abnormally distributed continuous data). Chi-square tests were applied for comparisons of categorical data. Variables for which the *P* value was < 0.05 in the univariate analysis were assessed by Cox regression analysis to evaluate the independent predictors of 36-month primary and secondary endpoints and the results are shown as hazards ratios (HRs) with 95% confidential intervals (CIs). Model 2 was adjusted for age and gender. Model 3 was built to control for confounders and adjusted for age, sex, heart rate, BMI, LVDd, LM disease, chronic total occlusion, diffuse lesion, target vessel of LM, complete revascularization, number of stents, total bilirubin, direct bilirubin, TC, triglyceride, glucose, platelet, ARB, sacubitril/valsartan, metformin, and SGLT2 inhibitor. The incidence rates of MACE and its components among MHR quartiles were calculated by the Kaplan‒Meier method and verified by the log-rank test. Subgroup analysis was developed to determine the influence of the MHR on MACE and its components in hypercholesterolemia and nonhypercholesterolemia patients. Spearman correlation was used to analysis the association between MHR and conventional risk factor. Nonlinear correlations between MHR and risk of MACE were demonstrated by restricted cubic spline models based on the Cox proportional hazards model with four knots (5th, 35th, 65th, and 95th percentiles of MHR) based on the lowest value of the Akaike information criterion. The adjusted variables in the model were consistent with Model 3.

## Results

In total, 1049 patients with a mean age of 61.4 ± 10.7 years (75.5% male) were enrolled in this study. The median follow-up was 36 months (IQR 18–36 months). Any missing data on outcomes, monocyte count, and HDL-C were deleted. Patients were divided into four quartiles based on MHR levels (MHR 0.26 ± 0.05 in Quartile 1, 0.40 ± 0.03 in Quartile 2, 0.54 ± 0.05 in Quartile 3, and 0.84 ± 0.22 in Quartile 4). According to the MHR quartiles, the baseline demographics, vital signs, BMI, comorbidities, medical history, NYHA class, echocardiography, angiographic data, procedural results, laboratory findings, and medication use are shown in Table 1. Apart from monocytes, HDL-C, and MHR, we also found that sex, SBP, BMI, FBG, D-dimer, INR, TC, triglycerides, ALT, AST, GGT, uric acid, potassium, calcium, white blood cells, platelets, hs-CRP, loop diuretics, sulfonylurea, and DDP4 depressor showed differences in MHR quartiles.

**Table 1:** Clinical, angiographic, haematologic, and medical history characteristics of the ischaemic heart failure patient combined with diabetes undergoing percutaneous coronary intervention according to monocyte to high-density lipoprotein cholesterol ratio

Characteristics	Total (*n* = 1049)	Quartiles of MHR				*P* value
		Quartile 1 (*n* = 265) MHR < 0.33 (× 10^9^/mmol)	Quartile2 (*n* = 261) 0.33 ≤ MHR < 0.46 (× 10^9^/mmol)	Quartile3 (*n* = 261) 0.46 ≤ MHR < 0.62 (× 10^9^/mmol)	Quartile4 (*n* = 262) 0.62 ≥ MHR (× 10^9^/mmol)	
Gender, *n* (%)						
Male	813 (75.5)	222 (61.7)	280 (77.8)	311 (86.4)	224 (85.5)	** < 0.001**
Female	267 (24.7)	138 (38.3)	80 (22.2)	49 (13.6)	38 (14.5)	
Age (years)	61.4 ± 10.7	62.2 ± 9.7	61.7 ± 10.9	60.9 ± 10.2	60.7 ± 11.9	0.307
Vital sigh						
SBP (mmHg)	124.8 ± 18.6	127.3 ± 18.6	124.5 ± 20.2	124.8 ± 18.5	122.4 ± 16.8	**0.028**
DBP (mmHg)	73.3 ± 12.1	74.0 ± 13.0	72.7 ± 12.0	74.2 ± 12.5	72.1 ± 10.7	0.114
HR (bpm)	74.4 ± 10.3	74.4 ± 10.3	73.5 ± 10.9	74.8 ± 10.7	74.9 ± 11.3	0.418
Body mass index(kg/m^2^)	28.9 ± 1.0	29.4 ± 0.9	29.1 ± 0.7	28.8 ± 0.7	28.2 ± 1.3	** < 0.001**
Comorbidities, *n* (%)						
Hypertension	714 (68.1)	186 (70.2)	168 (64.4)	179 (68.6)	181 (69.1)	0.503
Hypercholesterolemia	793 (75.6)	189 (71.3)	196 (75.1)	199 (76.3)	209 (79.8)	0.158
Renal insufficiency	510 (48.6)	134 (50.6)	128 (49.0)	122 (46.7)	126 (48.1)	0.845
Atrial fibrillation	57 (5.4)	14 (5.3)	15 (5.8)	17 (6.5)	11 (4.2)	0.698
History, *n* (%)						
Prior MI	250 (23.8)	68 (25.7)	60 (23.0)	62 (23.8)	60 (22.9)	0.869
Prior PCI	124 (11.8)	30 (11.3)	34 (13.0)	34 (13.0)	26 (9.9)	0.638
Prior stroke	134	33	38	27	36	0.499
NYHA class, *n* (%)						
I	102 (9.7)	29 (10.9)	24 (9.2)	30 (11.5)	19 (7.3)	0.626
II	576 (54.9)	143 (54.0)	147 (56.3)	145 (55.6)	141 (53.8)	
III	332 (31.7)	87 (32.8)	79 (30.3)	77 (29.5)	89 (34.0)	
IV	39 (3.7)	6 (2.3)	11 (4.2)	9 (3.5)	13 (3.7)	
Echocardiography						
LVDs (millimetre)	39.9 ± 7.8	39.5 ± 7.3	40.5 ± 8.0	39.5 ± 8.0	40.1 ± 7.9	0.414
LVDd (millimetre)	53.9 ± 6.8	53.3 ± 6.2	54.4 ± 7.2	53.9 ± 7.0	53.9 ± 6.9	0.375
LVEF (%)	44.7 ± 9.5	44.9 ± 9.1	44.3 ± 9.4	45.9 ± 9.9	43.7 ± 9.1	0.054
Angiographic data, *n* (%)						
LM disease	197 (18.8)	43 (16.2)	54 (20.7)	47 (18.0)	53 (20.2)	0.528
Three‑vessel disease	611 (58.3)	141 (53.2)	159 (60.9)	156 (59.8)	155 (59.2)	0.289
Chronic total occlusion	295 (28.1)	67 (25.3)	77 (29.5)	73 (28.0)	78 (29.8)	0.646
Diffuse lesion	205 (19.5)	49 (18.5)	45 (17.2)	55 (21.1)	56 (21.418)	0.570
In-stent restenosis	55 (5.2)	18 (6.8)	12 (4.6)	13 (5.0)	12 (4.6)	0.622
SYNTAX score	22.0 ± 7.9	21.0 ± 7.1	22.4 ± 8.4	22.2 ± 7.6	22.5 ± 8.3	0.121
Procedural results						
Target vessel territory, *n* (%)						
LM	177 (16.9)	39 (14.7)	49 (18.8)	44 (16.9)	45 (17.2)	0.667
LAD	779 (74.3)	195 (73.6)	200 (76.6)	189 (72.4)	195 (74.4)	0.730
LCX	685 (65.3)	171 (64.5)	166 (63.6)	164 (62.8)	184 (70.2)	0.271
RCA	710 (67.7)	175 (66.0)	185 (70.9)	184 (70.5)	166 (63.4)	0.192
Complete revascularization, *n* (%)	624 (59.5)	165 (62.3)	162 (62.1)	148 (56.7)	149 (56.9)	0.369
Number of stents	3.3 ± 1.5	3.3 ± 1.4	3.4 ± 1.4	3.4 ± 1.6	3.3 ± 1.5	0.571
Laboratory parameters						
Glucose (mmol/L)	9.0 ± 3.5	8.5 ± 3.2	9.1 ± 3.5	9.1 ± 3.4	9.3 ± 3.7	0.057
HbA1c (%)	7.9 ± 1.4	7.8 ± 1.5	7.9 ± 1.4	7.9 ± 1.4	8.0 ± 1.4	0.457
GA (%)	20.7 ± 5.2	20.7 ± 5.3	20.7 ± 5.3	20.7 ± 5.3	20.6 ± 5.1	0.990
BNP (pg/ml)	334 (144,507)	308 (135,478)	300 (144,487)	337 (150,484)	373 (147,577)	0.164
FBG (mg/dL)	3.6 ± 1.0	3.4 ± 0.7	3.5 ± 0.8	3.6 ± 1.0	3.9 ± 1.2	** < 0.001**
D-dimer	138 (87,204)	138 (83,193)	138 (84,191)	138 (81,212)	138 (103,226)	**0.014**
INR	1.0 ± 0.1	1.0 ± 0.1	1.0 ± 0.1	1.0 ± 0.1	1.1 ± 0.1	**0.008**
TC (mmol/L)	4.0 ± 1.1	4.02 ± 1.1	4.0 ± 1.1	3.9 ± 1.1	3.9 ± 1.1	**0.005**
Triglyceride (mg/dL)	1.8 ± 1.3	1.7 ± 1.2	1.8 ± 1.3	1.9 ± 1.1	2.1 ± 1.6	**0.003**
LDL-C (mmol/L)	2.4 ± 0.9	2.4 ± 1.0	2.4 ± 0.9	2.4 ± 0.9	2.3 ± 0.8	0.223
HDL-C (mmol/L)	1.0 ± 0.2	1.2 ± 0.3	1.0 ± 0.2	0.9 ± 0.2	0.8 ± 0.2	** < 0.001**
Total bilirubin (mg/dL)	0.8 ± 0.3	0.8 ± 0.3	0.7 ± 0.3	0.8 ± 0.4	0.7 ± 0.3	0.635
Direct bilirubin (μmol/L)	4.1 ± 2.0	3.8 ± 1.9	4.2 ± 2.1	4.0 ± 2.0	4.2 ± 2.1	0.099
ALT (U/L)	21 (15,32)	21 (14,29)	21 (14,31)	22 (14,31)	23 (15,38)	**0.012**
AST (U/L)	20 (16,30)	20 (16,27)	19 (15,26)	20 (16,30)	24 (16,44)	**0.002**
ALP (U/L)	78.4 ± 24.7	79.5 ± 24.5	78.5 ± 28.7	78.9 ± 23.0	77.0 ± 22.2	0.686
GGT (U/L)	30.7 ± 17.4	28.8 ± 17.0	31.0 ± 18.0	33.6 ± 19.1	29.5 ± 14.8	**0.009**
Creatinine (mg/dL)	1.0 ± 0.9	1.0 ± 1.0	1.0 ± 0.9	1.0 ± 0.8	1.1 ± 1.0	0.305
Blood nitrogen urea (mg/dL)	7.1 ± 3.5	7.0 ± 2.9	7.1 ± 4.1	7.1 ± 3.5	7.2 ± 3.6	0.967
eGFR (mL/min × 1.73 m^2^)	82.9 ± 24.5	83.2 ± 23.4	83.8 ± 24.5	83.9 ± 22.6	80.9 ± 27.2	0.455
Uric acid (μmol/L)	362.5 ± 104.5	340.6 ± 92.6	358.6 ± 100.4	372.0 ± 112.3	379.0 ± 108.2	** < 0.001**
Sodium (mmol/L)	138.8 ± 3.0	138.9 ± 2.9	139.1 ± 3.1	138.8 ± 2.8	138.7 ± 3.2	0.487
Potassium (mmol/L)	4.2 ± 0.5	4.2 ± 0.4	4.3 ± 0.5	4.2 ± 0.4	4.1 ± 0.5	**0.013**
Calcium (mmol/L)	2.3 ± 0.1	2.3 ± 0.1	2.3 ± 0.1	2.3 ± 0.1	2.3 ± 0.1	** < 0.001**
Magnesium (mmol/L)	0.9 ± 0.1	0.9 ± 0.1	0.9 ± 0.1	0.9 ± 0.1	0.9 ± 0.1	0.105
White blood cell (10^9^/L)	7.8 ± 2.4	6.6 ± 2.0	7.3 ± 1.7	7.9 ± 1.8	9.4 ± 3.0	** < 0.001**
Haemoglobin (g/dL)	134.8 ± 19.4	132.4 ± 19.8	135.3 ± 18.8	136.0 ± 19.2	135.5 ± 19.5	0.135
Monocyte (10^9^/L)	0.5 ± 0.2	0.3 ± 0.1	0.4 ± 0.1	0.5 ± 0.1	0.7 ± 0.2	** < 0.001**
Platelet (10^9^/L)	221.5 ± 66.0	210.8 ± 67.1	215.3 ± 62.0	220.6 ± 61.3	239.6 ± 69.7	** < 0.001**
hs-CRP	6.1 ± 7.9	3.3 ± 5.5	4.7 ± 6.9	6.8 ± 8.0	9.9 ± 9.0	** < 0.001**
MHR (10^9^/mmol)	0.51 ± 0.25	0.26 ± 0.05	0.40 ± 0.03	0.54 ± 0.05	0.84 ± 0.22	** < 0.001**
Medication use, *n* (%)						
Aspirin	1043 (99.4)	265 (100.0)	259 (99.2)	258 (98.9)	261 (99.6)	0.335
Clopidogrel	869 (82.8)	224 (84.5)	222 (85.1)	213 (81.6)	210 (80.2)	0.385
Ticagrelor	180 (17.2)	41 (15.5)	39 (14.9)	48 (18.4)	52 (19.9)	0.385
Statins	1038 (99.0)	265 (100.0)	256 (98.1)	258 (98.9)	259 (98.9)	0.191
Ezetimibe	268 (25.6)	58 (21.9)	73 (28.0)	71 (27.2)	66 (25.2)	0.382
ACEI	90 (8.6)	26 (9.8)	21 (8.1)	16 (6.1)	27 (10.3)	0.308
ARB	147 (14.0)	37 (14.0)	28 (10.7)	48 (18.4)	34 (13.0)	0.081
Beta-blockers	641 (61.1)	151 (57.0)	150 (57.5)	165 (63.2)	175 (66.8)	0.060
CCB	200 (19.1)	52 (19.6)	55 (21.1)	55 (21.1)	38 (14.5)	0.176
Diuretics						
Loop diuretics	630 (60.1)	150 (56.6)	146 (55.9)	147 (56.3)	187 (71.4)	** < 0.001**
Thiazide diuretics	61 (5.8)	20 (7.6)	13 (5.0)	17 (6.5)	11 (4.2)	0.352
Spironolactone	494 (47.1)	124 (46.8)	118 (45.2)	127 (48.7)	125 (47.7)	0.878
Tolvaptan	31 (3.0)	7 (2.6)	8 (3.1)	4 (1.5)	12 (4.6)	0.225
Sacubitril/valsartan	368 (35.1)	85 (32.1)	97 (37.2)	86 (33.0)	100 (38.2)	0.367
Oral hypoglycemic agents						
Metformin	268 (25.6)	54 (20.4)	64 (24.5)	72 (27.6)	78 (29.8)	0.075
Alpha‑glucosidase inhibitor	195 (18.6)	54 (20.4)	45 (17.2)	46 (17.6)	50 (19.1)	0.783
Sulfonylurea	55 (5.2)	21 (7.9)	14 (5.4)	6 (2.3)	14 (5.3)	**0.038**
SGLT2 inhibitor	402 (38.3)	98 (37.0)	104 (39.9)	101 (38.7)	99 (37.8)	0.142
DDP4 depressor	70 (6.7)	11 (4.2)	16 (6.1)	15 (5.8)	28 (10.7)	**0.019**
Insulin	402 (38.3)	101 (38.1)	96 (36.8)	96 (36.8)	109 (41.6)	0.632
Oral anticoagulants						
Warfarin	20 (1.9)	4 (1.5)	5 (1.9)	6 (2.3)	5 (1.9)	0.932
Factor Xa inhibitors	17 (1.6)	6 (2.3)	5 (1.9)	3 (1.2)	3 (1.2)	0.667
Factor IIa inhibitors	10 (1.0)	3 (1.1)	4 (1.5)	2 (0.8)	1 (0.4)	0.568

The normal distributed continuous variables are presented as mean ± SD. The abnormal distributed continuous variables are presented as median (25th, 75th). Categorical variables were presented as number (percentage). *P* values were calculated using analysis of variance. ANOVA (for normal distributed continuous data), Kruskal–Wallis (for abnormal distributed continuous data), and Chi-square (for categorical data) tests were used to compare differences in variables between different MHR quantiles

*P* < 0.05 (in bold)

SBP, systolic blood pressure; DBP, dilated blood pressure; HR, heart rate; MI, myocardial infarction; PCI, percutaneous coronary intervention; NYHA, New York Heart Association; LVDs, Left ventricular end systolic diameter; LVDd, Left ventricular end diastolic diameter; LVEF, left ventricular injection fraction; LM, left main artery; LAD, left anterior descending artery; LCX, left circumflex artery; RCA, right coronary artery; HbA1c, glycosylated haemoglobin A1c; GA, Glycated albumin; BNP, B-natriuretic peptide; FBG, fibrinogen; INR, International normalized ratio; TC, total cholesterol; LDL-C, low-density lipoprotein cholesterol; HDL-C, high-density lipoprotein cholesterol; ALT, alanine transaminase; AST, aspartate transaminase; ALP, alkaline phosphatase; GGT, gamma-glutamyl transferase; eGFR, estimated glomerular filtration rate; hs-CRP, high-sensitivity C-reactive protein; MHR, monocyte to high-density lipoprotein cholesterol ratio; ACEI, angiotensin-converting enzyme inhibitor; ARB, angiotensin receptor blocker; CCB, calcium channel blocker; GLP1, glucagon-like peptide 1; SGLT2, sodium-glucose cotransporter 2; DDP4, dipeptidyl peptidase 4 inhibitor

The MACE was 62 (23.4%) in MHR Quartile 1, 94 (36.0%) in Quartile 2, 108 (41.4%) in Quartile 3, and 143 (54.6%) in Quartile 4. The incidence of MACE was highest in the MHR Quartile 4 group, followed by the Quartiles 3, 2, and 1 groups (*P* < 0.001). The all-cause mortality event and incidence were 31 (11.7%) in Quartile 1, 47 (18.0%) in Quartile 2, 54 (20.7%) in Quartile 3, and 63 (24.1%) in Quartile 4. The incidence of all-cause mortality was highest in the MHR Quartile 4 group, followed by the MHR Quartiles 3, 2, and 1 (*P* = 0.003). The events and incidence of any revascularization showed a similar trend [63 (24.1%), 42 (16.1%), 38 (14.6%), and 26 (9.8%) in Quartiles 4–1, respectively, *P* < 0.001]. However, among these groups, no significant difference was noted in the incidence of nonfatal MI (1.9%, 3.5%, 4.6%, and 6.5% in Quartiles 1, 2, 3, and 4, respectively, *P* = 0.056) (Table 2).

**Table 2:** Outcomes of patients stratified by MHR quartiles

Outcomes	Total (*n* = 1049)	Quartiles of MHR				*P* value
		Quartile 1 (*n* = 265) MHR < 0.33 (× 10^9^/mmol)	Quartile 2 (*n* = 261) 0.33 ≤ MHR < 0.46 (× 10^9^/mmol)	Quartile 3 (*n* = 261) 0.46 ≤ MHR < 0.62 (× 10^9^/mmol)	Quartile 4 (*n* = 262) 0.62 ≤ MHR (× 10^9^/mmol)	
MACE, *n* (%)	407 (38.8)	62 (23.4)	94 (36.0)	108 (41.4)	143 (54.6)	< 0.001
All-cause mortality	195 (18.6)	31 (11.7)	47 (18.0)	54 (20.7)	63 (24.1)	0.003
Nonfatal MI	43 (4.1)	5 (1.9)	9 (3.5)	12 (4.6)	17 (6.5)	0.056
Any revascularization	169 (16.1)	26 (9.8)	38 (14.6)	42 (16.1)	63 (24.1)	< 0.001

Categorical variables were presented as number (percentage)

MHR, monocyte to high-density lipoprotein cholesterol ratio; MACE**,** major adverse cardiovascular events; MI, myocardial infarction

In Cox regression analyses, independent predictors of MACE (Table 3) were assessed. The MHR Quartile 1 was defined as the reference. No variables were adjusted in Model 1. Age and sex were adjusted for Model 2. The variables with *P* < 0.05 in stepwise regression were adjusted for Model 3, and these variables included age, sex, heart rate, BMI, LVDd, LM disease, chronic total occlusion, diffuse lesion, SYNTAX score, target vessel of LM, complete revascularization, number of stents, total bilirubin, direct bilirubin, TC, triglyceride glucose, platelet, ARB, sacubitril/valsartan, metformin, and SGLT2 inhibitor. In Models 1 and 2, the MHR was proven to be an independent risk factor for MACE, all-cause mortality, nonfatal MI, and any revascularization. Model 3, after adjusting for other variables, indicated that the MHR was an independent predictor for MACE (adjusted HR: 2.11; 95% CI 1.47–3.03; *P* < 0.001). The MHR was also an independent risk factor for all the defined components of MACE [for all-cause mortality (adjusted HR: 1.94 95% CI 1.14–3.29; *P* = 0.015), nonfatal MI (adjusted HR: 4.53 95% CI 1.66–12.39; *P* = 0.003), and any revascularization (adjusted HR: 1.93 95% CI 1.10–3.39; *P* = 0.023) (Table 3).

**Table 3:** The association between MHR and MACE

	Model 1			Model 2			Model 3		
	HR (95% CIs)	*P*	*P* for trend	HR (95% CIs)	*P*	*P* for trend	HR (95% CIs)	*P*	*P* for trend
MACE			< 0.001			< 0.001			< 0.001
Quartile 1: MHR < 0.33	1.0 (Ref)			1.0 (Ref)			1.0 (Ref)		
Quartile 2: 0.33 ≤ MHR < 0.46	1.66 (1.20–2.29)	0.002		1.66 (1.20–2.30)	0.002		1.59 (1.15–2.22)	0.006	
Quartile 3: 0.46 ≤ MHR < 0.62	2.00 (1.47–2.74)	< 0.001		2.04 (1.48–2.80)	< 0.001		1.89 (1.37–2.62)	< 0.001	
Quartile 4: MHR ≥ 0.62	2.82 (2.09–3.80)	< 0.001		2.83 (2.09–3.29)	< 0.001		2.29 (1.66–3.18)	< 0.001	
Continuous	3.03 (2.20–4.18)	< 0.001		1.74 (2.08–3.85)	< 0.001		2.11 (1.47–3.03)	< 0.001	
All-cause mortality			< 0.001			< 0.001			0.003
Quartile 1: MHR < 0.33	1.0 (Ref)			1.0 (Ref)			1.0 (Ref)		
Quartile 2: 0.33 ≤ MHR < 0.46	1.66 (1.06–2.61)	0.028		1.69 (1.07–2.67)	0.026		1.66 (1.04–2.66)	0.035	
Quartile 3: 0.46 ≤ MHR < 0.62	2.02 (1.30–3.14)	0.002		2.07 (1.32–3.26)	0.002		1.98 (1.25–3.13)	0.003	
Quartile 4: MHR ≥ 0.62	2.50 (1.63–3.85)	< 0.001		2.56 (1.65–3.99)	< 0.001		2.05 (1.28–3.29)	0.003	
Continuous	2.85 (1.78–4.56)	< 0.001		2.80 (1.74–4.50)	< 0.001		1.94 (1.14–3.29)	0.015	
Nonfatal MI			0.002			0.004			0.006
Quartile 1: MHR < 0.33	1.0 (Ref)			1.0 (Ref)			1.0 (Ref)		
Quartile 2: 0.33 ≤ MHR < 0.46	1.97 (0.66–5.89)	0.223		1.91 (0.63–5.76)	0.253		1.79 (0.58–5.48)	0.308	
Quartile 3: 0.46 ≤ MHR < 0.62	2.76 (0.97–7.85)	0.056		2.68 (0.93–7.75)	0.068		2.53 (0.86–7.42)	0.091	
Quartile 4: MHR ≥ 0.62	4.16 (1.53–11.29)	0.005		3.99 (1.44–11.09)	0.008		3.90 (1.35–11.28)	0.012	
Continuous	4.56 (1.85–11.23)	0.001		1.54 (1.15–2.05)	0.004		4.53 (1.66–12.39)	0.003	
Any revascularization			< 0.001			< 0.001			0.001
Quartile 1: MHR < 0.33	1.0 (Ref)			1.0 (Ref)			1.0 (Ref)		
Quartile 2: 0.33 ≤ MHR < 0.46	1.60 (0.97–2.63)	0.066		1.58 (0.95–2.62)	0.077		1.52 (0.91–2.54)	0.108	
Quartile 3: 0.46 ≤ MHR < 0.62	1.85 (1.14–3.03)	0.013		1.87 (1.13–3.07)	0.014		1.70 (1.02–2.81)	0.040	
Quartile 4: MHR ≥ 0.62	2.94 (1.86–4.65)	< 0.001		2.91 (1.82–4.65)	< 0.001		2.31 (1.40–3.81)	0.001	
Continuous	2.90 (1.75–4.79)	< 0.001		2.72 (1.64–4.51)	< 0.001		1.93 (1.10–3.39)	0.023	

Models were derived from Cox proportional hazards regression analysis. *Model 1*: unadjusted. *Model 2*: adjusted for age, sex. *Model 3*: adjusted for age, sex, heart rate, body mass index, LVDd, LM disease, chronic total occlusion, diffuse lesion, target vessel of LM, complete revascularization, number of stents, total bilirubin, direct bilirubin, TC, triglyceride glucose, platelet, ARB, sacubitril/valsartan, metformin, SGLT2 inhibitor. HR, hazards ratio; CI, confidence interval; MACE**,** major adverse cardiovascular events; Ref, as reference; MHR, monocyte to high-density lipoprotein cholesterol ratio; MI, myocardial infarction; LVDd, Left ventricular end diastolic diameter; LM, left main artery; TC, total cholesterol; ARB, angiotensin receptor blocker; SGLT2, sodium-glucose cotransporter 2

**Fig. 2:**
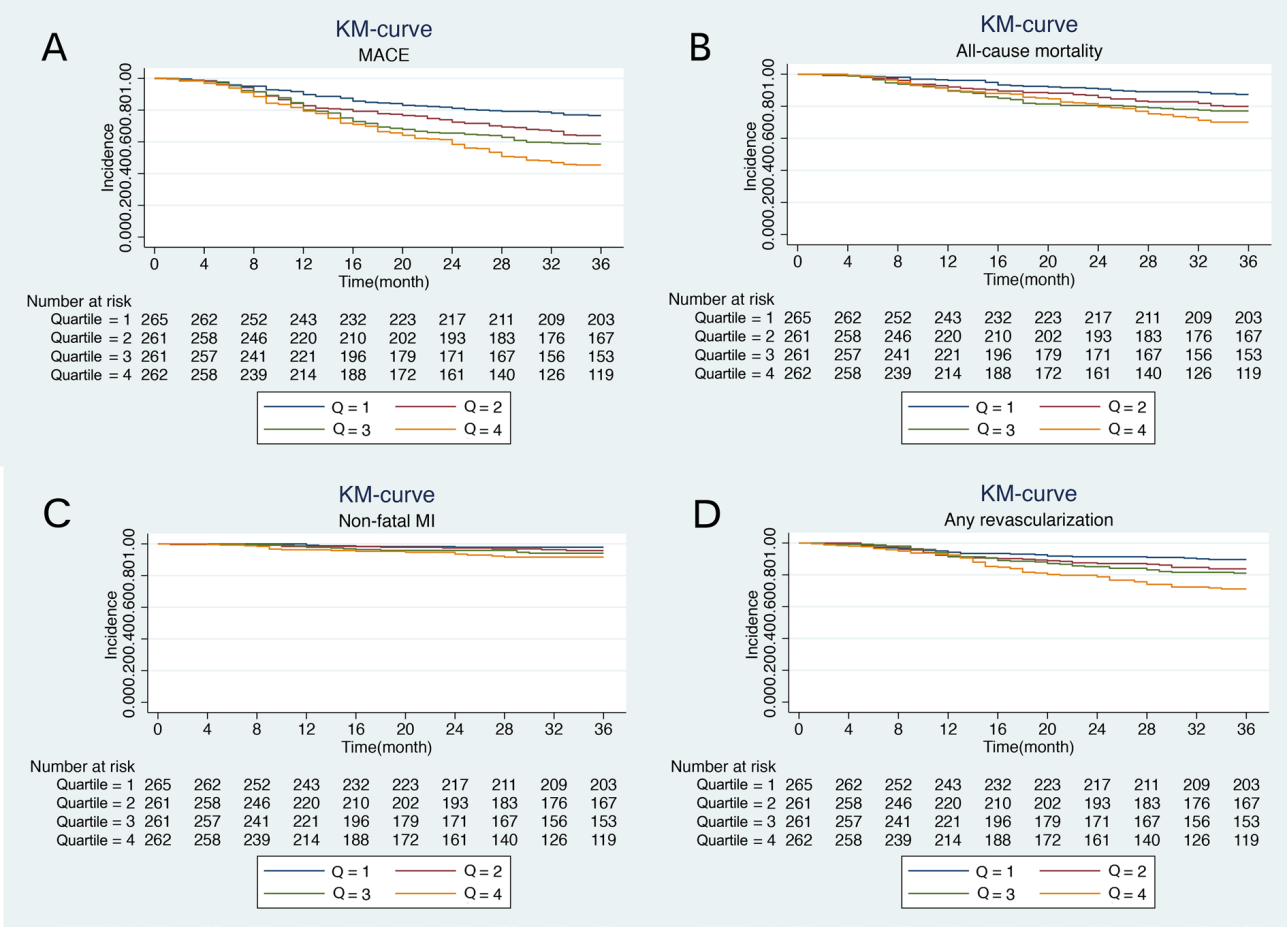
Kaplan‒Meier analysis. Kaplan‒Meier survival curves showing the incidence of major adverse cardiovascular events, all-cause mortality, nonfatal myocardial infarction, and any revascularization among ischaemic heart failure patients with diabetes mellitus. The curves were stratified into 4 quartiles by different levels of monocyte to high-density lipoprotein cholesterol ratio (MHR). The deep blue line is for Quartile 1 (Q1), crimson for Quartile 2 (Q2), green for Quartile 3 (Q3), and orange for Quartile 4 (Q4). Log-rank test *P* < 0.05 for each

Kaplan‒Meier analysis according to MACE-free survival revealed a higher occurrence of MACE in MHR Quartile 4, followed by Quartiles 3, 2, and 1 (*P* < 0.0001) (Fig. 2A). Kaplan‒Meier analysis according to all-cause mortality-free survival revealed the highest occurrence of all-cause mortality in the MHR Quartile 4 group, followed by Quartiles 3, 2, and 1 (*P* = 0.0002) (Fig. 2B). Kaplan‒Meier curves based on the stratified values of MHR also showed that the patients with a higher MHR were found to have a significantly higher nonfatal-MI probability and revascularization probability (*P* = 0.001 and *P* < 0.0001) (Fig. 2C, D).

**Fig. 3:**
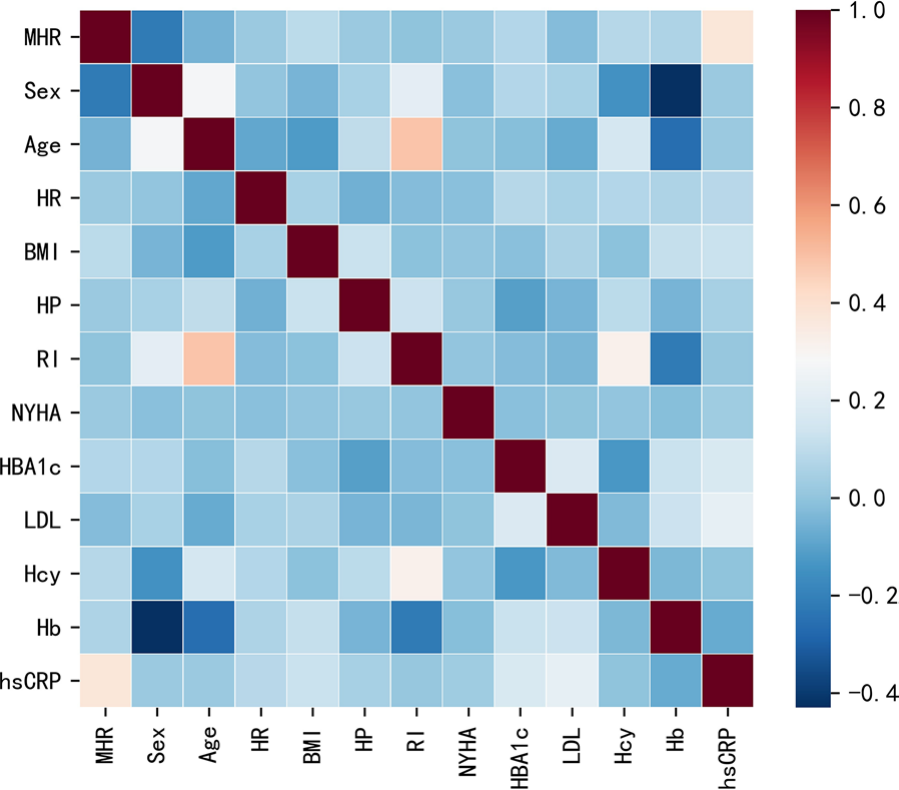
Heatmap of Spearman correlations analysis. Spearman correlations between MHR and conventional risk factors for MACE. MHR, monocyte to high-density lipoprotein cholesterol ratio; MACE, major adverse cardiovascular events; HR, heart rate; BMI, body mass index; HP, hypertension; RI, renal insufficiency; NYHA, New York Heart Association; HBA1c, haemoglobin A1c; LDL-C, low-density lipoprotein cholesterol; Hcy, homocysteine; Hb, Haemoglobin; hs-CRP, high-sensitivity C-reactive protein

Previous studies have established both hypercholesterolemia and the MHR as risk factors for MACE. To explore whether there are potential interaction effects between hypercholesterolemia and MHR, we conducted interaction relationship analysis based on Cox regression. We found no significant interaction effect between hypercholesterolemia and MHR (*P* for interaction > 0.05 in Models 1, 2, and 3). The existence of hypercholesterolemia did not significantly affect the association between MHR and MACE among the subjects in Models 1, 2, and 3. The results of the subgroup analysis are shown in Table 4. We also made further investigation into correlations between MHR and known risk factors of MACE. Spearman correlation analysis showed that MHR was positively correlated with BMI (*r* = 0.094, *P* = 0.003), HbA1c (*r* = 0.074, *P* = 0.018), homocysteine (*r* = 0.081, *P* = 0.009), haemoglobin (*r* = 0.065, *P* = 0.038), and hs-CRP (*r* = 0.374, *P* < 0.001), but negatively correlated with gender female (*r* = − 0.220, *P* < 0.001). No correlations were found between MHR levels and age, heart rate, hypertension, renal insufficiency, NYHA class, and LDL-C (Table 5). The heatmap of Spearman correlation coefficients is shown in Fig. 3.

**Table 4:** The interaction effects between MHR and MACE in patients with hypercholesterolemia or no hypercholesterolemia

Hypercholesterolemia	HR (95% CI)	*P* Value	No hypercholesterolemia	HR (95% CI)	*P* Value	*P* for interaction
Model 1			Model 1			0.867
Quartile 1: MHR < 0.33	Reference		Quartile 1: MHR < 0.33	Reference		
Quartile 2: 0.33 ≤ MHR < 0.46	1.45 (1.00–2.09)	0.051	Quartile 2: 0.33 ≤ MHR < 0.46	2.46 (1.28–4.71)	0.007	
Quartile 3: 0.46 ≤ MHR < 0.62	1.73 (1.20–2.47)	0.003	Quartile 3: 0.46 ≤ MHR < 0.62	3.11 (1.65–5.86)	< 0.001	
Quartile 4: MHR ≥ 0.62	2.66 (1.90–3.72)	< 0.001	Quartile 4: MHR ≥ 0.62	3.19 (1.66–6.11)	< 0.001	
Continuous	1.37 (0.67–2.82)	0.389	Continuous	2.94 (1.51–5.73)	0.001	
Model 2			Model 2			0.963
Quartile 1: MHR < 0.33	Reference		Quartile 1: MHR < 0.33	Reference		
Quartile 2: 0.33 ≤ MHR < 0.46	1.48 (1.02–2.16)	0.041	Quartile 2: 0.33 ≤ MHR < 0.46	2.23 (1.15–4.31)	0.017	
Quartile 3: 0.46 ≤ MHR < 0.62	1.78 (1.24–2.57)	0.002	Quartile 3: 0.46 ≤ MHR < 0.62	2.82 (1.45–5.46)	0.002	
Quartile 4: MHR ≥ 0.62	2.74 (1.94–3.89)	< 0.001	Quartile 4: MHR ≥ 0.62	2.93 (1.52–5.65)	0.001	
Continuous	1.33 (0.65–2.72)	0.431	Continuous	2.55 (1.30–5.02)	0.007	
Model 3			Model 3			0.803
Quartile 1: MHR < 0.33	Reference		Quartile 1: MHR < 0.33	Reference		
Quartile 2: 0.33 ≤ MHR < 0.46	1.46 (0.99–2.14)	0.053	Quartile 2: 0.33 ≤ MHR < 0.46	2.26 (1.09–4.69)	0.029	
Quartile 3: 0.46 ≤ MHR < 0.62	1.61 (1.11–2.33)	0.011	Quartile 3: 0.46 ≤ MHR < 0.62	3.38 (1.62–7.09)	0.001	
Quartile 4: MHR ≥ 0.62	2.17 (1.50–3.13)	< 0.001	Quartile 4: MHR ≥ 0.62	3.29 (1.49–7.27)	0.003	
Continuous	1.17 (0.55–2.50)	0.687	Continuous	1.86 (0.79–4.35)	0.153	

Models were derived from Cox proportional hazards regression analysis. **Model 1**: unadjusted. **Model 2**: adjusted for age, sex. **Model 3**: adjusted for age, sex, heart rate, body mass index, LVDd, LM disease, chronic total occlusion, diffuse lesion, target vessel of LM, complete revascularization, number of stents, total bilirubin, direct bilirubin, TC, triglyceride glucose, platelet, ARB, sacubitril/valsartan, metformin, SGLT2 inhibitor

HR, hazards ratio; CI, confidence interval; MACE**,** major adverse cardiovascular events; MHR, monocyte to high-density lipoprotein cholesterol ratio; MI, myocardial infarction; LVDd, left ventricular end diastolic diameter; LM, left main artery; TC, total cholesterol; ARB, angiotensin receptor blocker; SGLT2, sodium-glucose cotransporter 2

**Table 5:** Association between MHR and conventional risk factors

Conventional risk factors for MACE	Correlation coefficient with MHR	95% CI	*P*
MHR	1.000	1.000 to 1.000	** < 0.001**
Gender	− 0.220	− 0.277 to − 0.157	** < 0.001**
Age	− 0.052	− 0.113 to 0.010	0.098
HR	0.018	− 0.043 to 0.083	0.563
BMI	0.094	0.033 to 0.155	**0.003**
Hypertension	0.021	− 0.041 to 0.086	0.494
RI	− 0.003	− 0.065 to 0.057	0.923
NYHA class	0.022	− 0.042 to 0.084	0.482
HbA1c	0.074	0.015 to 0.136	**0.018**
LDL-C	− 0.025	− 0.084 to 0.039	0.423
Homocysteine	0.081	0.014 to 0.144	**0.009**
Haemoglobin	0.065	− 0.003 to 0.124	**0.038**
hs-CRP	0.374	0.317 to 0.428	** < 0.001**

The correlation coefficients between MHR and the conventional risk factors of MACE were assessed by Spearman correlation analysis

*P* < 0.05 (in bold)

MHR, monocyte to high-density lipoprotein cholesterol ratio; MACE**,** major adverse cardiovascular events; CI, confidence interval; HR, heart rate; BMI, body mass index; RI, renal insufficiency; NYHA, New York Heart Association; HbA1c, haemoglobin A1c; LDL-C, low-density lipoprotein cholesterol; hs-CRP, high-sensitivity C-reactive protein

**Fig. 4:**
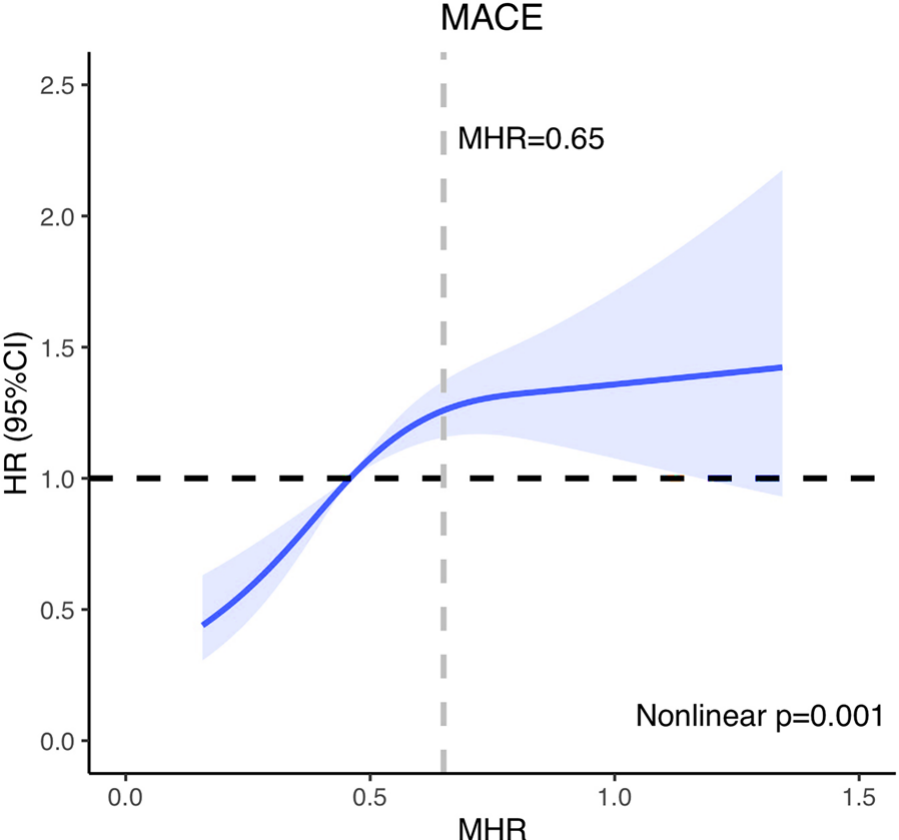
Restricted cubic spline analysis. Multivariable adjusted hazards ratio for major adverse cardiovascular events according to the monocyte/high-density lipoprotein cholesterol ratio (MHR) on a continuous scale. The adjusted variables are age, sex, heart rate, body mass index, left ventricular end diastolic diameter, left main disease, chronic total occlusion, diffuse lesion, target vessel of left main artery, complete revascularization, number of stents, total bilirubin, direct bilirubin, total cholesterol, triglyceride glucose, platelet, angiotensin receptor blocker, sacubitril/valsartan, metformin, and sodium-dependent glucose transporters 2 inhibitor. The solid blue line represents the multivariable adjusted hazards ratio, and the light blue area represents the 95% confidence interval. The cut-off point of the MHR is 0.65

The restricted cubic spline curve based on Cox proportional hazards models was used to visualize the nonlinear relation of MHR with MACE. MHR showed an overall positive relationship with the risk of MACE in ischaemic HF patients with DM undergoing PCI. The curve increased rapidly with a steep slope until an MHR of approximately 0.65 was reached. When the MHR was higher than 0.65, the slope was relatively flat. This represented a significant increase in MACE risk when the MHR was lower than 0.65. The risk of MACE increased slowly when the MHR exceeded 0.65. Therefore, the MHR might have higher discrimination for the risk of MACE when the MHR was less than 0.65, while the discrimination was limited when the MHR had a higher value (Fig. 4).

## Discussion

To the best of our knowledge, this is the first study to implicate the MHR in an ischaemic HF cohort with DM. Furthermore, the MHR was also first proposed to be a predictor of MACEs in ischaemic HF patients with concomitant DM undergoing PCI. A higher MHR value was found to have a higher incidence of MACE. It was also determined that the MHR was an independent predictor of MACE in ischaemic HF patients with DM undergoing PCI and had no interaction with hypercholesterolemia.

The mortality of ischaemic HF has increased in recent decades by 20.6% [37, 38]. CAD is the leading cause of this condition [1]. It is well acknowledged that DM and CAD are homogeneous or equivalent. Additionally, DM and HF independently increase the risk for the other [4]. Meanwhile, most of them have received PCI treatment. Therefore, the population of ischaemic HF combined with DM undergoing PCI is an underestimated large group. Although all-cause chronic HF combined with DM has been widely investigated, the population of specific ischaemic HF combined with DM undergoing PCI has been investigated.

MHR, deprived from routine test items, is monocyte count divided by HDL-C. It has emerged as a marker for systemic oxidative stress and inflammation [23]. Monocytes have chemotaxis effects. Activated monocytes with oxidized LDL and other lipids transform into foam cells, taking an essential part in plaque formation, which leads to the deteriorating development of ACS and MACE [39]. In addition, activated monocytes differentiate into macrophages and trigger the release of various inflammatory cytokines in chronic heart diseases [40, 41]. In contrast, HDL-C has antioxidant and anti-inflammatory effects [42–44]. HDL-C was able to inhibit monocyte activation, adhesion, migration, and LDL-C oxidation [45, 46]. Murphy et al. found that HDL-C participated in the formation of apolipoprotein, which inactivated CD1b and acted on its anti-inflammatory effects [42]. In addition, HDL-C is also a well-known protective factor for cardiovascular disease [47]. Therefore, through the ratio of “oxidative and inflammatory promotion” and “oxidative and inflammatory inhibition”, the increasing MHR value represents the severity of inflammation, hence indicating exacerbations of HF, aggravated progression, and a higher incidence of MACE. In addition, the MHR also partially reflected the greater burden of comorbidities, such as DM, hypertension, obesity, and renal insufficiency, in patients with HF [30]. These comorbidities also caused a higher incidence of MACEs.

MHR as a prognostic factor for MACE after PCI in CAD has been investigated before. There are some studies consistent with ours. In patients with ACS undergoing PCI, the prognostic effects of the MHR have been demonstrated [20]. In patients with ST‐segment elevation myocardial infarction (STEMI) undergoing primary PCI who had elevated MHR values, the incidences of in-hospital or long-term MACE and the rates of short or late mortality were significantly higher [48–50]. In non-ST‐segment elevation myocardial infarction patients undergoing PCI, the MHR was also an independent risk factor for in-hospital MACE with an OR of 8.36, which exhibited similar predictive performance to STEMI [24]. Additionally, the MHR was associated with increased MACE risk after an adjusted HR of 2.734 in unstable angina patients scheduled for selective PCI [51]. These studies as well as our study indicate that the MHR has a close relationship with the CAD spectrum. MHR is also related to many cardiovascular disorders combined with DM. Previous studies have explored cardiovascular disorders combined with DM cohorts, such as non-ST-segment elevation acute coronary syndrome [24], subclinical left cardiac remodelling and dysfunction [30], and arterial stiffness [31]. All of them showed that the MHR is associated with the occurrence and outcome of certain cardiovascular disorders. However, the application value of the MHR in an ischaemic HF combined with DM cohort has never been investigated before. Therefore, our study filled the research gap and provided a rational background for the clinical management of this population.

Epidemiological research indicates a high prevalence of hypercholesterolemia in patients with concurrent ischaemic HF and DM. Previous studies have established both hypercholesterolemia and MHR as risk factors for adverse prognosis in these patients. Therefore, this study investigated the potential relationship between hypercholesterolemia and MHR. Hypercholesterolemia was identified as a confounding factor in the stepwise regression analysis. However, even after adjusting for hypercholesterolemia, MHR remained an independent risk factor for MACE in Model 3. Furthermore, there was no significant difference in the predictive ability of MHR between the subgroup of patients with hypercholesterolemia and the subgroup without hypercholesterolemia. These findings suggest that MHR possesses distinct underlying mechanisms for predicting MACE compared to hypercholesterolemia. Overall, hypercholesterolemia did not compromise the independent predictive capacity of MHR for MACE, as evidenced by both the subgroup analysis and multivariable regression analysis. These findings further strengthen the robustness of MHR in predicting MACE. However, prospective prespecified subgroup analysis is still necessary to validate the reliability of MHR and assess potential influences from other variables.

In Model 3, some adjusted variables were related to the comorbidities of obesity, abnormal liver function, hyperlipidaemia, and DM, such as body mass index, total bilirubin, direct bilirubin, TC, triglyceride, fast blood glucose, metformin, and SGLT2 inhibitor. They partly contribute to the incidence of MACE. Notably, other adjusted variables related to the severity of coronary artery lesions and the results of PCI procedures, such as left main artery disease, chronic total occlusion, diffuse lesion, target vessel of left main artery, complete revascularization, and number of stents, suggested that severe lesions had negative impacts on ischaemic HF and its prognosis, especially for lesions significantly reducing the myocardial blood supply, such as left main artery disease, chronic total occlusion, and diffuse lesion. In contrast, complete revascularization ameliorated these impacts. These findings are consistent with the aetiology and pathophysiology of ischaemic HF, as well as a previous study showing that the values of MHR were positively related to myocardial infarct sizes [19].

In our study, we found that the MHR may be a stronger predictor of MACE at MHR values lower than 0.65 in the restricted cubic spline model. One possible explanation is that the restricted cubic spline in this study was based on Model 3 and adjusted for several confounders, including well-established factors known to influence MACE, such as age, sex, TC, BMI, SGLT2 inhibitor, and sacubitril/valsartan. Adjusting for these factors diminished the predictive capacity of MHR for MACE, with this reduction being more pronounced at higher MHR values. This observation suggests that MHR is more susceptible to the influence of confounding factors at higher values. Therefore, caution should be exercised when applying MHR in practice, especially when it exhibits an exceptionally high value.

Additionally, we conducted comparisons of the discrimination ability of the MHR for incidence of MACE between cohorts of ischaemic HF with and without DM. Following the same exclusion criteria as illustrated in Fig. 1, we identified 2584 eligible cases for analysis in the ischaemic HF cohort. Kaplan–Meier curves were generated to illustrate the incidence rates of MACE in different MHR quartiles within the ischaemic HF cohort. The MHR exhibited superior discrimination in predicting observed outcomes in the ischaemic HF cohort with DM compared to the ischaemic HF cohort alone (see Fig. 2 and Additional file 1: Fig. S1). These findings suggest that MHR performs well in patients with ischaemic HF and DM, indicating that this particular cohort may derive greater benefit from the predictive ability of MHR.

There are some limitations in this study. Given that this study is a single-centre study, the generalizability of the conclusions derived from this research to other centres or regions may be limited. Further data from additional centres are necessary in the future to enhance the robustness and applicability of our findings. Due to the characteristics of retrospective studies, the results might have been affected by recall deviation, especially medication use. Besides, we were unable to further compare the predictive role of MHR for MACE in ischaemic HF cohort and ischaemic HF with DM cohort because of the incomprehensive clinical baseline data in ischaemic HF cohort.

## Conclusion

In conclusion, a high MHR is an independent predictor of MACE, all-cause mortality, nonfatal MI, and any revascularization at 36 months in ischaemic HF combined with DM patients who have undergone PCI, suggesting that the MHR might be a promising predictor for identifying this population with a higher risk of poor clinical outcomes after PCI treatment.

### Supplementary Information

**Additional file 1**: **Fig. S1**. Kaplan–Meier analysis. Kaplan–Meier survival curves showing the incidence of major adverse cardiovascular events, all-cause mortality, nonfatal myocardial infarction, and any revascularization among the ischaemic heart failure patients. The curves were stratified by 4 quartiles by different levels of monocyte to high-density lipoprotein cholesterol ratio (MHR). Deep blue line is for Quartile 1, crimson for Quartile 2, green for Quartile 3, and orange for Quartile 4.

## Data Availability

The datasets used and analysed during the current study are available from the corresponding author on reasonable request.

## Abbreviations

ACEI: Angiotensin-converting enzyme inhibitor
ACS: Acute coronary syndrome
ALT: Aspartate transaminase
ARB: Angiotensin receptor blocker
AST: Alkaline phosphatase
BMI: Body mass index
BNP: B-natriuretic peptide
CAD: Coronary artery disease
CI: Confidential interval
DDP4: Dipeptidyl peptidases inhibitor 4
DM: Diabetes mellitus
eGFR: Estimated glomerular filtration rate
FBG: Fibrinogen
GGT: Gamma-glutamyl transferase
HDL-C: High-density lipoprotein cholesterol
HF: Heart failure
hs-CRP: High-sensitivity C-reactive protein
HR: Hazards ratio
INR: International normalized ratio
LAD: Left anterior descending artery
LCX: Left circumflex artery
LDL-C: Low-density lipoprotein cholesterol
LM: Left main artery
LVDd: Left ventricular end diastolic dimension
LVDs: Left ventricular end systolic dimension
LVEF: Left ventricular ejection fraction
MACE: Major adverse cardiovascular events
MHR: Monocyte/HDL-C ratio
MI: Myocardial infarction
NHR: Neutrophil to high-density lipoprotein ratio
NLR: Neutrophil to lymphocyte ratio
NYHA: New York Heart Association
PCI: Percutaneous coronary intervention
RCA: Right coronary artery
SGLT2: Sodium-dependent glucose transporters 2
STEMI: ST‐segment elevation myocardial infarction
SYNTAX: Synergy between PCI with taxus and cardiac surgery
TC: Total cholesterol
