# Supplementary figures and images for: Monocyte to high-density lipoprotein cholesterol ratio predicts poor outcomes in ischaemic heart failure patients combined with diabetes: a retrospective study
Source: Eur J Med Res. 2023 Nov 8;28:493. doi: 10.1186/s40001-023-01451-6 (PMC10631131; doi:10.1186/s40001-023-01451-6)

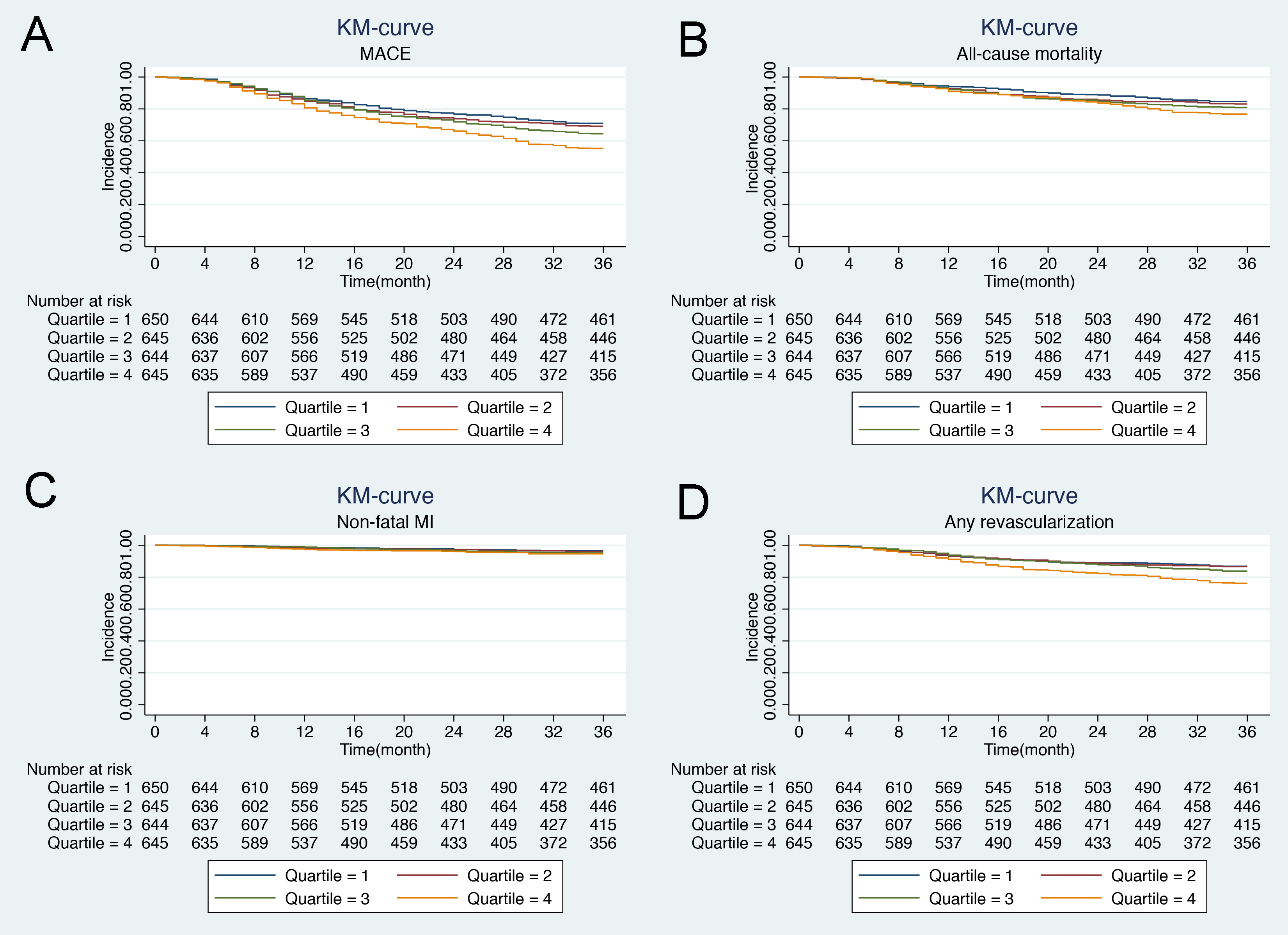

Supplement: Supplementary file 1 — Additional file 1: Fig. S1. Kaplan–Meier analysis. Kaplan–Meier survival curves showing the incidence of major adverse cardiovascular events, all-cause mortality, nonfatal myocardial infarction, and any revascularization among the ischaemic heart failure patients. The curves were stratified by 4 quartiles by different levels of monocyte to high-density lipoprotein cholesterol ratio (MHR). Deep blue line is for Quartile 1, crimson for Quartile 2, green for Quartile 3, and orange for Quartile 4. [file 40001_2023_1451_MOESM1_ESM.jpg]
